# Influence of efavirenz pharmacokinetics and pharmacogenetics on neuropsychological disorders in Ugandan HIV-positive patients with or without tuberculosis: a prospective cohort study

**DOI:** 10.1186/1471-2334-13-261

**Published:** 2013-06-04

**Authors:** Jackson K Mukonzo, Alphonse Okwera, Neoline Nakasujja, Henry Luzze, Deogratious Sebuwufu, Jasper Ogwal-Okeng, Paul Waako, Lars L Gustafsson, Eleni Aklillu

**Affiliations:** 1 Division of Clinical Pharmacology, Department of Laboratory Medicine, Karolinska Institutet, Karolinska University Hospital Huddinge, SE- 141 86, Stockholm, Sweden; 2 Department of Pharmacology & Therapeutics, College of Health Sciences, Makerere University, Kampala, Uganda; 3 Department of Internal Medicine, College of Health Sciences, Makerere University, Kampala, Uganda; 4 Department of Psychiatry, College of Health Sciences, Makerere University, Kampala, Uganda; 5 School of Public Health, College of Health Sciences, Makerere University, Kampala, Uganda

**Keywords:** Efavirenz, Neuropsychiatric toxicity, Rifampicin, CYP2B6, HIV, Tuberculosis, CNS, Antiretroviral therapy, Ugandans

## Abstract

**Background:**

HIV infection, anti-tuberculosis and efavirenz therapy are associated with neuropsychological effects. We evaluated the influence of rifampicin cotreatment, efavirenz pharmacokinetics and pharmacogenetics on neuropsychiatric disorders in Ugandan HIV patients with or without tuberculosis coinfection.

**Methods:**

197 treatment naïve Ugandan HIV patients, of whom 138 were TB co-infected, enrolled prospectively and received efavirenz based HAART. TB-HIV confected patients received concomitant rifampicin based anti-TB therapy. Genotypes for *CYP2B6* (*6, *11), *CYP3A5* (*3, *6, *7), ABCB1 (c.3435C>T and c.4036 A/G rs3842), *CYP2A6* (*9, *17) and *NR1I3* rs3003596 T/C were determined. Efavirenz plasma concentrations were serially quantified at 3rd day, 1st, 2nd, 4th, 6th, 8th and 12th weeks during therapy. Efavirenz neuropsychiatric symptoms were evaluated in terms of sleep disorders, hallucinations and cognitive effects at baseline, at two and twelve weeks of efavirenz treatment using a modified Mini Mental State Examination (MMSE) score.

**Results:**

During the first twelve weeks of ART, 73.6% of the patients experienced at least one efavirenz related neuropsychiatric symptom. Commonest symptoms experienced were sleep disorders 60.5% (n=124) and hallucination 30.7% (n=63). Neuropsychiatric symptoms during HAART were significantly predicted by efavirenz plasma concentrations consistently. Rifampicin cotreatment reduced plasma efavirenz concentrations significantly only during the first week but not afterwards. There was no significant difference in the incidence of neuropsychiatric symptoms between patients receiving efavirenz with or without rifampicin cotreatment. CYP2B6*6 and ABCB1 c.4036 A/G genotype significantly predicted efavirenz concentrations. The tendency of CYP2B6*6 genotype association with higher incidence of having vivid dream (p=0.05), insomnia (p=0.19) and tactile hallucination (p=0.09) was observed mainly at week-2.

**Conclusions:**

Efavirenz related neuropsychiatric symptoms are common among Ugandan HIV patients receiving ART and is mainly predicted by higher efavirenz plasma concentrations and *CYP2B6* genotype but not by rifampicin based anti-TB co-treatment.

## Background

The severity of HIV-associated neurocognitive disorders has been reduced in the era of HAART but neurological effects due to antiretroviral toxicity have become an increasing burden in HIV-infected patients [1,2]. Despite the indisputable benefits, concerns with the adverse effects of efavirenz use particularly neuropsychiatric symptoms are well recognized [3-5]. Efavirenz crosses the blood–brain barrier attaining cerebral spinal fluid concentrations 0.5 to 1.2% of its corresponding plasma concentrations, reaching therapeutic concentrations in brain [6,7]. Clinical studies have reported high rates of neuropsychiatric side effects including vivid dreams, insomnia and mood changes in > 50% of the patients who initiate efavirenz [8-12]. Although most CNS symptoms are mild, up to one-fifth of all individuals commencing efavirenz-containing regimen discontinue the drug and switch therapy primarily because of unbearable neurotoxicity, minimizing further therapeutic options [13].

Efavirenz is primarily metabolized to 8-hydroxyefavirenz mainly by CYP2B6 and to a lesser extent by CYP3A [14,15]. Importance of CYP3A in mediating efavirenz metabolism particularly in subjects with CYP2B6 slow metabolizers is described recently [16]. CYP2A6 mediated 7-hydroxylation accounts about 23% of total efavirenz metabolism [15]. P-glycoprotein, encoded by ABCB1, is the major efflux transporter at the blood–brain barrier that limits entry into the CNS for a large number of drugs, and probably contributes to patient-to-patient variability in response to CNS pharmacotherapy [17]. Although in vitro and animal studies report that P-glycoprotein is not the main cellular transporter protein for efavirenz [18], several studies in African HIV patients reported association of genetic variation in ABCB1 with efavirenz plasma/intracellular concentrations and/or treatment outcome. Associations between ABCB1 c.4036A>G with higher plasma efavirenz concentrations in Ugandan HIV patients was first reported by our group [19], a finding latter confirmed in South African [20], Ethiopian and Tanzanians [21] as well as in other non-African populations [22]. Association of ABCB1 c.3435C>T variation with CD4-cell recovery after efavirenz therapy initiation is also reported [22,23]. Accordingly P-glycoprotein may have a role in efavirenz cellular transport or alternatively the functional genetic variants in ABCB1 might be in strong linkage disequilibrium with other SNPs located in another gene relevant for efavirenz disposition and hence may serve as tag SNP. All enzymes involved in efavirenz disposition including ABCB1 are inducible by efavirenz mainly via the activation of human constitutive androstane receptor (hCAR), encoded by *NR1I3* gene [24]. Several studies reported association of genetic variation in *CYP2B6*, *CYP3A5*, *CYP2A6*, *ABCB1* and *NR1I3* with plasma efavirenz concentration in HIV patients is reported [16,19,20,25,26]. However effects of pharmacogenetic variations on susceptibility to efavirenz induced neuropsychiatric disorders particularly in Sub-Saharan African HIV patients remains to be investigated well.

Mid-dose or trough efavirenz plasma concentrations <1 μg/mL is reported to increase the risk of virologic failure, while concentrations > 4 μg/mL increased likelihood of developing neuropsychiatric side effects in HIV-infected patients [27,28]. Rifampicin, a potent inducer of drug metabolizing enzymes and transporter proteins, reduces efavirenz plasma concentration but the extent of drug interaction varies between populations and is influenced by *CYP2B6* genotype [29-33]. Treatment success with efavirenz therefore requires maintenance of an optimal plasma concentration ensuring a balance between neuropsychiatric toxicity and possible treatment failure [34]. Complex drug interactions might also contribute to CNS symptoms and this might be particularly relevant in patients being treated with efavirenz-containing regimens. CNS side effects associated with other first line anti tuberculosis drugs such as isoniazid [35-38] may further complicate TB-HIV co-treatment.

Clinical studies reported increased levels of efavirenz and higher rates of neuropsychiatric disorders particularly in patients of African origin [39,40]. A recent study reported association of black ethnicity and CYP2B6 *516TT* genotype with early discontinuation of efavirenz-containing regimens [41]. The defective *CYP2B6 G516T*variant allele, which impairs efavirenz metabolism, is common in African populations [16,19] and its impact on efavirenz plasma concentrations may surpass the counter effect of rifampicin co-administration [31-33]. Variations in efavirenz tolerability during rifampicin treatment may therefore be attributed to pharmacogenetic factors that influence neuropsychiatric toxicity during TB-HIV co-treatment.

While the role of pharmacogenetics in efavirenz – rifampicin pharmacokinetic interactions has been substantively investigated, the possibly compounded neuropsychiatric toxicities during co- treatment with the two drugs have not been adequately described. Because of high prevalence of HIV, efavirenz based HAART is widely used in Sub-Saharan Africa, but data on the incidence and risk factors of efavirenz associated neuropsychiatric symptoms are limited [42,43]. African populations display wide genetic heterogeneity and hence data from one geographic region may not be extrapolated directly to others with in the continent [44,45] and hence more studies are required to evaluate the incidence and risk factors of efavirenz related neuropsychiatric disorders. In the present study we evaluated pharmacogenetic and pharmacokinetic aspects of efavirenz associated Neuropsychological symptoms in Ugandan HIV patients receiving efavirenz based HAART with or without rifampicin based anti-tuberculosis therapy. The primary objective was to evaluate the incidence, timing, predictors and type of efavirenz based HAART associated neuropsychiatric disorders in Ugandan HIV patients with or without tuberculosis coinfection. Our secondary objectives were to evaluate 1) Effect of rifampicin co-treatment on the incidence of efavirenz based HAART associated neuropsychiatric disorders 2) Association between neuropsychiatric disorders and efavirenz plasma concentrations monitored on day 3 and at 1, 2, 4, 6, 8 and 12 weeks during therapy. 3) Associations of pharmacogenetic variations in *CYP2B6*, *CYP3A5*, *CYP2A6*, *ABCB1* and *NR1I3* with susceptibility to efavirenz induced neuropsychiatric disorders.

## Methods

Newly diagnosed ART naïve HIV patients with or without tuberculosis coinfection attending the HIV/TB or HIV clinic at Mulago and Butabika National referral Hospitals in Kampala Uganda were recruited and enrolled into the study during the years 2008 to 2009. HIV only infected patients received efavirenz based HAART as per the national treatment guideline. HIV-TB coinfected patients received rifampicin based anti-TB regimen for 6 months under direct observe therapy (2 months ethambutol / isoniazid / rifampicin /pyrazinamide followed by 4 months of isoniazid and rifampicin combination therapy) initiated 2–8 weeks before ART. Adherence to ART was evaluated based on self-report. Fifty-nine of the patients were HIV infected without TB co-infection and received ART therapy only. CD4 analysis was performed to ensure eligibility for HIV treatment. Only patients eligible for efavirenz according to the national treatment guidelines at enrolment were recruited into the study. All eligible patients were treated, for HIV, with efavirenz 600mgs daily in combination with zidovudine and lamivudine and followed up for at least 6 months. Blood samples for genotyping were collected at enrollment while mid-dose efavirenz plasma concentration samples for the study were collected during follow-up visits. Patients were evaluated for central neuropsychiatric disorders at baseline and on follow-up visits at weeks 2 and 12. The study was approved by local ethical committees at Mulago and Butabika hospitals and the Uganda National Council for Science and Technology. A written informed consent was obtained from each study participant.

### Efavirenz pharmacokinetic analysis

Blood samples were collected into EDTA tubes at enrollment and at 11 to 18 hours post dose on day 3 and at weeks 1, 2, 4, 6, 8 and 12. Plasma was prepared from blood samples by centrifugation at 3000 rpm for 10 min and stored at -70°C until HPLC analysis was performed. Plasma efavirenz was determined by reverse phase HPLC with UV-detection as previously described [19]. The HPLC machine, Agilent series 1100, consisting of column compartment G1316A, Degasser G132A, Quat pump G1311A, and an auto-sampler ALS, G1329A, and G1315B diode array detector was used. The column used was Ace3C18, 3 μm 50×30 mm (Advanced Chromatography Technologies, Aberdeen, Scotland). The standard used was efavirenz (99.9), supplied by the WHO Collaborating center for chemical reference substances through Apoteket AB Stockholm, Sweden. The retention time for efavirenz was 2.42 minutes as detected at UV–VIS 1, 210 nm, UV–VIS 2, 220 nm. Within-day coefficient of variations was 3.2, 3.3 and 5.1 at concentrations of 2.0 μM (n=17), 8.0 μM (n=17), and 20 μM (n=16) respectively and between-day coefficient of variation was 4.1 (n=50).

### Genotyping

Genomic DNA was isolated from peripheral blood leukocytes using QIAamp DNA Maxi Kit (QIAGEN GmbH. Hilden. Germany). All participants were genotyped for CYP2B6*6 and *11, CYP3A5*3,*6 and *7 and ABCB1 (3435CT and rs3842).Allelic discrimination reactions were performed using TaqMan (Applied Biosystems, CA, USA) genotyping assays: (C___7586657_20 for *ABCB1* 3435C>T, C___7817765_60, for *ABCB1* rs3842T>C, C__29560333_20, for *CYPB6* 516G>T [CYP2B6*6 ], for *CYP2B6* 136A>G [CYP2B6*11], C__26201809_30 for *CYP3A5* 6986A>G [CYP3A5*3], C_16194070_10 for *NR1I3* rs3003596T/C, C_30634332_10 for *CYP2A6 -48T>G* [CYP2A6*9], C_34816076_20 for *CYP2A6 5065 G>A* [CYP2A6*17], C__30203950_10 for *CYP3A5* 14690G>A [CYP3A5*6]) and C__32287188_10 for *CYP3A5* g.27131_27132insT [CYP3A5*7] on ABI 7500 FAST (Applied Biosystems, Foster City, CA). The final volume for each reaction was 10μl, consisting of 2x TaqMan Universal PCR Master Mix (Applied Biosystems, Foster City, CA, USA). 20 × drug metabolising genotype assay mix and 10 ng genomic DNA. The PCR profile consisted of an initial step at 50°C for 2 min and 50 cycles with 95°C for 10 minutes and 92°C for 15 sec.

### Neuropsychiatric symptom monitoring

Efavirenz neuropsychiatric effects were evaluated in terms of sleep disorders, hallucinations and cognitive effects at baseline, two and twelve weeks of efavirenz treatment. The adjusted mini mental status evaluation (MMSE) form that takes care of the possible differences due to variations in the level of education and age differences [46] was supplemented with questions which were used to assess sleep disorders and halucinations.

Assessment for sleep disorders was based on patient’s experience of insomnia, vivid dreams and sleepwalking as far as they could remember at baseline and during the first 2 weeks and 2 to 12 weeks of ART initiation. Patients were declared to have experienced mild insomnia if they failed to fall asleep within 15 minutes of going to bed, moderate insomnia if they went out of bed for up to one hour as a result of failure to sleep and severe insomnia if they went and stayed out of bed for more than one hour because of failure to sleep. Assessment of outcomes for both vivid dreams and sleepwalking were yes or no as declared by study participants. Overall assessment outcome for sleep disorder was no or yes for patients that did not or suffered any form of insomnia or experienced vivid dreams or sleep walking respectively. Patients were also assessed for three forms of hallucinations which included; auditory, visual and tactile hallucinations. The assessment outcome was no or yes for each form of hallucination and for hallucinations in general if the patient reported experience of any of the three forms. Patients were scored for cognition based only upon their responses to relevant questions according to the adjusted min mental status evaluation (MMSE) form [46]. Adjusted MMSE scores >23 were interpreted as normal while 20–23 and ≤19 were interpreted mild to moderate and severe cognitive disorders respectively. Overall, patients that either experienced any new or severer forms of neuropsychiatric symptoms following initiation of efavirenz were considered positive for the evaluation. For accuracy of data, a trained psychiatric nurse under the supervision of a physician administrated all neuropsychiatric assessment forms while completeness of data was improved by giving patients telephone reminders of their neuropsychiatric evaluation visits 24 hours in advance.

### Data analysis

Descriptive analysis was performed to determine the mean and the standard deviation for continuous variables and the percentages for categorical variables. Normality of efavirenz concentration data was achieved through log10 transformation of all values. Comparison of proportion of subjects with and without neuropsychiatric disorders between the two treatment groups, sex and genotype groups were done using Chi-square test. Correction for multiple testing was done using Bonferroni method. Comparisons of efavirenz plasma concentration between patients with or without neuropsychiatric disorders as well as between the two treatment groups (presence or absence of rifampicin cotreatment) were done using independent sample T-test. Levene’s test was applied to determine variance homogeneity. Multivariate analyses were conducted using logistic regression and linear mixed model to identify factors associated with neuropsychiatric symptoms. Efavirenz concentration, rifampicin treatment status and sex were included as predictors. Variables with p value < 0.3 in the Univariate analysis were included in the multivariate logistic regression with backward stepwise conditional elimination. Multivariate linear mixed effects model was used to control for the multiple repeated measurements contributed by the same patient at different time points. The dependent variable neuropsychiatric toxicity was code as yes =1 and no =0. Interaction test was performed, multi-collinearity problem checked and Hosmer and Lemeshaw test employed to examine the overall fitness of the model. Path analysis was performed to assess genotypes, rifampicin co-treatment and efavirenz concentrations as predictors for drug- associated neuropsychiatric toxicity. The level of statistical significance was specified at 0.05 for all tests. Statistical analysis was performed using IBM® SPSS® statistics software version 20.

## Results

A total of 197 subjects participated in the study. Fifty-nine of them were HIV patients treated with efavirenz based HAART only. The remaining 138 were HIV-TB confected patients treated with both efavirenz based HAART and rifampicin based anti-tuberculosis drugs. Demographic characteristics of study participants, measures of HIV disease and frequency of neuropsychiatric symptoms at baseline are presented in Table 1. Neuropsychiatric disorder was evaluated in 100%, 96% (98.6% for rifampicin and 91.5% for none rifampicin treatment groups) and 89.9% (94.9% for rifampicin and 80.5% for none rifampicin treatment groups) at baseline, week 2 and week 12 respectively.

**Table 1:** Baseline characteristics (demographics, measures of HIV disease and neuropsychiatric symptoms of HIV-only and HIV-TB co-infected (Rifampicin treatment) study participants at initiation of ART

**Baseline demographics and measures of HIV disease**	**All participants (n=197)**	**HIV- patients (n=59)**	**HIV-TB patients (n=138)**	**P-value**
Proportion of female participants	109 (55.3)	40 (67.8)	69 (50.4)	0.02
Body weight at ART initiation / kg (±SD)	53.6± 10.1	56.5 ± 9.8	51.9± 10.0	0.015
Age / years (±SD)	33.8± 7.2	36.5 ± 6.1	32.7±7.4	0.003
Initial CD4 count (±SD)	97.2± 77.4	123.1± 63.7	86.2± 79.9	0.009
Log_10_. viral load (±SD)	4.95± 0.71	4.81± 0.73	5.02± 0.70	0.08
**Baseline neuropsychiatric Symptoms**	61 (31)	14 (23.7 )	47 (34.1)	0.14
**Sleep disorders**	36 (18.3)	9(15.2)	27 (19.7)	0.46
*Insomnia*	21 (10.7)	6(10.2)	15(10.9)	0.88
*Vivid dreams*	11 (5.6)	4(6.9)	7(5.2)	0.66
*Sleep walking*	9 (4.6)	0(0.0)	9 (6.67)	0.04
**Hallucinations**	3(1.5)	1(1.7)	2(1.5)	0.9
*Auditory*	3(1.5)	1(1.7)	2(1.5)	0.9
*Visual*	0(0.0)	0(0.0)	0(0.0)	-
*Tactile*	0(0.0)	0(0.0)	0(0.0)	-
**Cognitive disorder**	46 (23.4)	9(15.2)	37 (27.0)	0.07

*SD* standard deviation.

### Neuropsychiatric disorders

Neuropsychiatric symptoms were overall reported in 31% of patients at ART initiation, the majority (74%) of whom exhibited a cognition disorder: MMSE score <19. Baseline neuropsychiatric symptoms did not significantly differ between HIV patients co-infected with or without TB except for cognitive disorder whose incidence was higher among patients co-infected with TB (Table 1).

During the initial twelve weeks of efavirenz treatment, 73.6% (n = 142) of the participants experienced at least one efavirenz related neuropsychiatric symptom. The commonest form of neuropsychiatric disorder exhibited was sleep disorders 60.5% (n= 124), followed by hallucination 30.7% (n = 63). Cognition improved with efavirenz based antiretroviral therapy, with the number of patients scored <19 on the MMSE being reduced from 46 (23.3%) at ART initiation to 22 (10.7%) at twelve weeks of treatment. The incidence of neuropsychiatric toxicity did not differ between the rifampicin and non-rifampicin treatment groups (p=0.70). Even at week two, when most neuropsychiatric symptoms were reported, no significant differences were observed between the two treatment groups.

Vivid dreams, the commonest sleep disorder symptom was experienced by 93% (n = 115) of those that suffered any form of efavirenz associated sleep disorder. All patients that experienced vivid dreams, except one, had them by second week of efavirenz treatment. Only 25% of 115 had persistent episodes of vivid dreams until week 12. Insomnia and sleepwalking were reported at rates of 36.3% (n= 45) and 8.1% (n= 10) respectively. About 89% (n= 40) of all patients that suffered from efavirenz related insomnia had symptoms by week two of treatment. 14 patients, among them 4 new cases, complained of insomnia at week 12 of efavirenz treatment. Almost all efavirenz related sleepwalking episodes occurred within two weeks after initiation of treatment. Only one patient reported sleepwalking after week two. The commonest type of hallucination was the audio form 75% (n= 47), followed by the visual 54% (n=34) and tactile 7.9% (n=5) forms. Two and 21 persons experienced all or at least two forms of hallucinations respectively. Like most of the other efavirenz neuropsychiatric symptoms, hallucinations occurred within the first two weeks of treatment, with only 10, 3 and 1 experiencing the audio, visual and tactile forms respectively, until week 12. The proportion of participants that suffered efavirenz neuropsychiatric toxicity did not significantly differ between women 76.8% (n=83) and men 69.4% (n=59) (p= 0.21), although overall, a tendency to exhibit higher efavirenz plasma concentrations among women at week-1 was observed (efavirenz plasma concentration _female_ =2.8 mgl^-1^versus efavirenz plasma concentration _male_ = 2.2 mgl^-1^, p=0.06). None of the study participants discontinued their treatment regimen because of severe neuropsychological problems.

#### Efavirenz plasma concentrations and Rifampicin cotreatment

Comparison of median efavirenz plasma concentrations between the two treatment groups monitored at each study time point is presented in Table 2. Significant differences in the mean plasma efavirenz concentrations between the two treatment groups (presence or absence of rifampicin cotreatment) were observed only during early initiation of efavirenz based HAART (at day-3 and week-1). Then after no significant differences in the mean plasma efavirenz concentration was observed.

**Table 2:** Comparison of median and interquartile range of plasma efavirenz (EFV) concentrations (mg/L) between HIV patients receiving EFV based HAART only and TB-HIV coinfected patients receiving EFV based HAART with rifampicin (RIF) based TB therapy

		**Treatment group**					**p**
		**EFV only**		**EFV + RIF**			
	**N**	**Median (IQR)**	**N**	**Median (IQR)**			
Day-3	57	2.48 (1.85-2.92)	107	1.85 (1.34-2.86)	0.02
week-1	53	2.37 (1.92-3.18)	57	1.80 (1.34-2.49)	0.01
week-2	53	2.21 (1.63-3.21)	100	1.94 (1.38-2.60)	0.21
week-4	49	2.45 (1.73-3.22)	103	1.87 (1.43-3.13)	0.48
week-6	38	2.37 (1.70-3.51)	86	1.89 (1.63-4.19)	0.93
week-8	50	2.41 (1.64-3.06)	118	1.82 (1.42-3.21)	0.40
week-12	44	2.41 (1.65-3.55)	101	2.04 (1.47-3.64)	0.69

Plasma efavirenz concentrations were monitored at day 3 and at 1, 2, 4, 6, 7, 8 and 12 weeks after starting efavirenz based HAART. For statistical analysis, Log transformed efavirenz concentration was used using independent sample T-test.

Comparison of plasma efavirenz concentrations between subjects who experienced neuropsychiatric disorders during therapy and who did not is presented in Table 3. There was significant association of having higher plasma efavirenz concentrations with experience of neuropsychiatric symptoms during efavirenz-based therapy. We also compared plasma efavirenz concentrations between the two treatment groups stratified by neuropsychiatric symptoms during therapy (Table 4). Interestingly among patients who experienced neuropsychiatric symptoms, the mean plasma efavirenz concentrations were comparable between the two treatment groups and no significant differences were observed.

**Table 3:** Comparison of median and interquartile range (IQR) of plasma efavirenz (EFV) concentrations (mg/L) between patients who presented with any neuropsychiatric disorders (Yes) and who did not (No) during efavirenz based HAART among Ugandan HIV patients with and without TB coinfection

		**Neuropsychiatric disorders**			**p**
		**No**		**Yes**	
	**N**	**Median (IQR)**	**N**	**Median (IQR)**	
Day-3	37	1.61 (1.37-2.29)	125	2.29 (1.52-2.92)	0.02
week-1	28	1.86 (1.43-2.55)	80	2.26 (1.67-3.1)	0.13
week-2	40	1.69 (1.32-2.15)	111	2.22 (1.41-3.21)	0.03
week-4	40	1.57 (1.31-2.25)	110	2.49 (1.58-3.57)	0.002
week-6	36	1.8 (1.42-2.61)	86	2.37 (1.66-4.25)	0.008
week-8	45	1.64 (1.36-2.81)	121	2.31 (1.53-3.21)	0.09
week-12	41	1.84 (1.45-2.57)	101	2.57 (1.68-4.19)	0.03

Plasma efavirenz concentrations were monitored at day 3 and at 1, 2, 4, 6, 7,8 and 12 weeks after starting efavirenz based HAART. For statistical analysis, Log transformed efavirenz concentration was used using independent sample T-test.

**Table 4:** Comparison of median and interquartile range of plasma efavirenz (EFV) concentrations (mg/L) between patients treated with EFV based HAART only (EFV only) versus TB-HIV coinfected patients treated with EFV based HAART plus RIF based TB therapy (EFV + RIF) stratified by observed neuropsychiatric symptoms during therapy (Yes) and who did not (No) during therapy

	**Neuropsychiatric disorders (No)**					**Neuropsychiatric disorders (Yes)**		
		**EFV only**	**EFV + RIF**			**EFV only**		**EFV + RIF**
	**N**	**Median (IQR)**	**N**	**Median (IQR)**	**N**	**Median (IQR)**	**N**	**Median (IQR)**
Day-3	15	2.29 (1.41-2.88)	22	1.48 (1.15-1.88)	41	2.6 (2.09-2.94)	84	1.97 (1.38-2.91)
week-1	13	2.25 (1.84-2.69)	15	1.53 (1.28-1.97)	39	2.47 (2.09-3.23)	41	1.9 (1.34-2.61)
week-2	15	1.9 (1.27-2.3)	25	1.66 (1.36-2.11)	37	2.51 (1.84-3.43)	74	2.05 (1.4-2.67)
week-4	12	1.64 (1.46-2.43)	28	1.57 (1.19-2.17)	36	2.65 (2.05-3.56)	74	2.37 (1.55-3.57)
week-6	12	2.11 (1.46-3.41)	24	1.77 (1.37-2.02)	25	2.43 (1.94-3.51)	61	2.17 (1.66-4.63)
week-8	14	1.69 (1.41-2.49)	31	1.56 (1.34-3.05)	35	2.48 (2.23-3.13)	86	1.91 (1.48-3.22)
week-12	12	1.86 (1.45-2.26)	29	1.84 (1.45-2.7)	31	2.61 (2.04-4.09)	70	2.29 (1.56-4.45)

No significant difference in EFV concentrations between treatment groups was observed.

Coherent with lack of rifampicin cotreatment on efavirenz plasma concentrations, there was no significant differences in the incidence of neuropsychiatric symptoms between HIV patients (72%) receiving EFV based HAART only versus TB-HIV coinfected patients (74%) receiving EFV based HAART with rifampicin based anti-tuberculosis therapy (chi square test p=0.73, odds ratio 1.126, 95% CI for odds ratio 0.56 to 2.25). Similarly logistic regression reviled no significant association of rifampicin cotreatment with Neuropsychiatric symptoms.

#### Effect of Pharmacogenetic variations

Efavirenz plasma concentrations were significantly influenced by *CYP2B6* genotypes at all study time points. The number of people having efavirenz plasma concentrations (>4 mg/l) increased from 14 on day 3 to 34 on day 84. Of the 14 and 34 people with high drug concentrations 6 and 12 were among the 9 and 13 people homozygous for CYP2B6*6 respectively. No significant effect of CYP2A6, *NR1I3* rs3003596, *CYP3A5* and *ABCB1* c.3435C>T genotype on efavirenz plasma concentrations was observed at all sampling occasions. *ABCB1 c.4036A>G* genotype was associated with having higher efavirenz plasma concentration at day-3, (p = 0.06) and week-8 (p=0.04). Effect of CYP2B6*11 genotype on efavirenz plasma concentration was observed at day-3 (p=0.07), week-2 (p=0.10) and week-4 (p=0.01).

**Figure 1:**
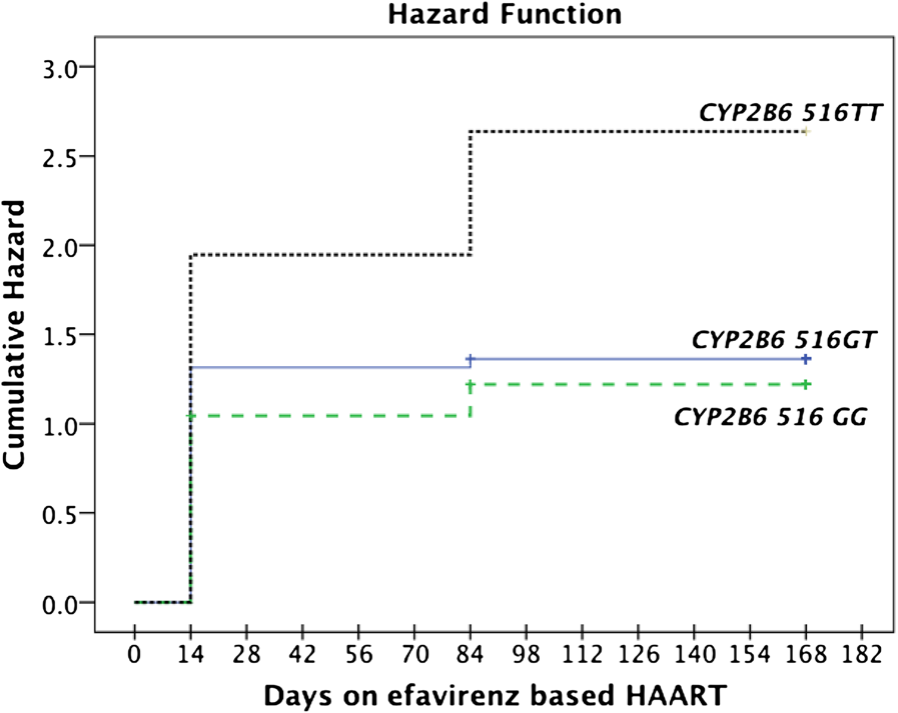
Kaplan–Meier curves indicating cumulative hazard for the development of efavirenz-based highly active antiretroviral therapy associated neuropsychiatric symptoms between the different CYP2B6 genotype groups during 12 weeks in Ugandan HIV patients.

Distribution of *CYP2B6*, *CYP2A6*, *CYP3A5*, *ABCB1* and *NR1I3* genotype and allele frequency between patients who devolved neuropsychiatric symptoms and who did not during efavirenz treatment is presented in Table 5. Neither *ABCB1* (c.4046A>G and c.3435C>T), CYP2A6 (*9 and *17) genotypes or the number of functional alleles in CYP3A5*1 examined in this study were related to neuropsychiatric toxicity. There was a tendency of CYP2B6*6 genotype association with higher incidence of having vivid dream (p=0.05), insomnia (p=0.19) and tactile hallucination (p=0.09) at week-2. Kaplan–Meier curves to estimate cumulative hazard for the development of efavirenz-based highly active antiretroviral related neuropsychiatric symptoms during 12 weeks follow-up period between the different *CYP2B6* genotype groups is presented in Figure 1. The proportion of patients with CYP2B6*6/*6 genotype was much higher (9.5%) among those with over all neuropsychiatric disorders compared to those without (2%). Almost all subjects with CYP2B6*6/*6 genotype except one experienced neuropsychiatric disorders. There was a tendency (p=0.06) of associations between *NR1I3* rs3003596CT genotype and neuropsychiatric symptoms (Table 5) but the difference was observed mainly between homozygous TT versus heterozygous CT genotype groups. Proportion of subject homozygous for rs3003596 C variant allele that is associated with low efavirenz plasma concentrations [20], were much higher (43.5%) among patients without neuropsychiatric symptoms compared those who experienced such symptom (24.3%). Univariate logistic regression analysis was done using each genotype, sex and treatment group as independent predictors. CYP2B6*6 genotype was associated with vivid dreams (p=0.05), sleepwalking (p=0.11), insomnia (p=0.19) and tactile hallucination (p=0.09). A tendency of CYP2B6*6 (0.21), NR1I3 rs3003596CT (0.06) genotype and sex (0.07) to predict the overall neuropsychiatric symptoms was observed. The predictive value did not change significantly in the multivariate analyses regression using backward conditional elimination and no significant interaction between CYP2B6*6, *NR1I3* rs3003596CT genotype and sex was observed.

**Table 5:** Genotype and allele frequency distributions among patients with or without neuropsychiatric disorders during efavirenz based HAART

**Genotype**		**Neuropsychiatric disorders**				**p value**
			**No**		**Yes**	
		**n**	**%**	**n**	**%**	
CYP2B6*6	*1/*1	21	42	61	44.5	0.16
	*1/*6	28	56	63	46	
	*6/*6	1	2	13	9.5	
CYP2B6*11	*1/*1	41	82	111	78.2	0.17
	*1/*11	8	16	31	21.8	
	*11/*11	1	2	0	0	
CYP2A6*9	*1/*1	39	84.8	98	79.7	0.51
	*1/*9	7	15.2	22	17.9	
	*9/*9	0	0	3	2.4	
CYP2A6*17	*1/*1	38	90.5	104	83.9	0.3
	*1/*17	3	7.1	19	15.3	
	*17/*17	1	2.4	1	0.8	
No. of CYP3A5*1 alleles^a^	*zero*	15	30	31	21.8	0.48
	*One*	24	48	79	55.6	
	*Two*	11	22	32	22.5	
ABCB1 (rs3842	*0/*0	29	63	87	65.4	0.95
	*0/*1	16	34.8	43	32.3	
	*1/*1	1	2.2	3	2.3	
ABCB1 c.3435C/T	*CC*	37	74	109	77.9	0.68
	*CT*	13	26	30	21.4	
	*TT*	0	0	1	0.7	
*NR1I3* rs3003569CT	*TT*	10	21.7	29	23	0.06
	*TC*	16	34.8	65	51.6	
	*CC*	20	43.5	32	25.4	
**Alleles**		**Frequency (%)**		**Frequency (%)**		
CYP2B6*6		30		32.5		0.21
CYP2B6*11		10		10.9		0.78
CYP2A6*9		7.61		11.38		0.31
CYP2A6*17		5.95		8.46		0.55
No. of CYP3A5*1 allele		46		50.4		0.56
ABCB1 rs3842		19.6		18.4		0.80
ABCB1 c.3435T		13		11.4		0.67
*NR1I3* rs3003569 T		39.1		48.8		0.11

P value from binary logistic regression is presented.

^a^ Haplotype analyses indicate no linkage between *CYP3A5* SNPs thus subjects were grouped based on number of functional *CYP3A5* allele (CYP3A5*1) for statistical analysis.

## Discussion

We examined the effects of plasma efavirenz concentrations, rifampicin cotreatment and pharmacogenetic variations in five relevant genes on the incidence of efavirenz related neuropsychiatric symptoms using sleep disorders, hallucinations and cognition as its measure. Our results indicate efavirenz plasma concentration as a main predictor of neuropsychiatric symptoms in Ugandan HIV patients receiving efavirenz based ART, a finding that is consistent with previous reports from other populations [28,34,42]. Efavirenz plasma concentrations were predicted by *CYP2B6* genotypes at all study time points. Though not statistically significant, we observed a tendency of CYP2B6*6 genotype to be associated with relatively higher incidence of sleep disorder and hallucination by second week of initiation of therapy. The cumulative hazards for the incidence of neuropsychiatric symptoms were relatively higher in CYP2B6*6 genotypes (Figure 1). Our result indicates that neither genetic polymorphism of *CYP3A5* and *ABCB1* (c.4036 A/G and C3435T), CYP2A6 (*9 and *17) nor rifampicin co-treatment are significantly correlated to efavirenz associated neuropsychiatric symptoms. We observed a tendency of NR1I3 rs3003596CT genotype and sex to be associated with neuropsychiatric symptoms. This study is one of the few that have examined efavirenz related neuropsychiatric toxicity during rifampicin treatment in an African context where HIV/TB co-infection and *CYP2B6* genetic variation are most prevalent.

HIV infection is characterized by acquired impairment in cognitive functioning, disturbances in memory, attention and processing speed [47]. It is important to note that HIV/TB co infected patients are usually very ill and hence their performance on cognitive tests may in part be influenced by their physical wellbeing. Interestingly one third of the study participants experienced neuropsychiatric symptoms, mainly cognitive disordered at baseline, which improved later during ART. Our result is supporting the evidence that HAART can improve the cognitive dysfunction [48,49].

On the other hand other neuropsychiatric symptoms associated with efavirenz therapy are recognized as a major cause of non-adherence [13]. Clinical studies have shown a high incidence of psychiatric effects among HIV patients being treated with efavirenz, ranging from 61% to 90% [10-12]. Similarly in the present study, 73% of the patients experienced neuropsychiatric symptoms during the initial twelve weeks of efavirenz treatment. Sleep disorders 60.5% were commonest followed by hallucination 30.7%.

Notable however, is the lack of any significant differences in the incidence of efavirenz related neuropsychiatric symptoms between the two treatment groups even at week two. Efavirenz product information indicates the possibility of 20% and 26% reduction in Cmax and AUC respectively, upon co-treatment with rifampicin [50]. Therefore rifampicin co-treatment is anticipated to lower plasma efavirenz concentration and hence lower incidence of neuropsychiatric symptoms among patient receiving concomitant therapy. But effect of rifampicin on efavirenz pharmacokinetics varies between populations. In the present study effect of rifampicin cotreatment on plasma efavirenz concentration was observed during the first week of efavirenz initiation. Then after there was no significant difference in the mean plasma efavirenz concentration between patients receiving efavirenz with or without rifampicin (Table 2). Coherent with this, we found comparable efavirenz related neuropsychiatric toxicity profiles among patients treated with or without rifampicin based anti-tuberculosis therapy, which is similar to other studies [51,52]. This finding is plausibly explained by similar efavirenz concentrations exhibited by patients in the two treatment groups. Similar to our finding, several studies mainly in black and Asian populations, reported insignificant effect of rifampicin on efavirenz plasma concentration or even paradoxically increased efavirenz concentration during rifampicin cotreatment [32,53-58]. Anti-TB drug adverse drug reactions have previously been reported to have significant psychological effects on patients [36,37,59]. This study however demonstrates that even with similar efavirenz plasma concentrations in both treatment groups, neuropsychiatric symptoms did not differ implying that anti-TB treatment related neuropsychiatric effects may not be significant among Ugandan HIV patients treated with efavirenz based regimens.

While this study demonstrates influence of *CYP2B6* genotype on efavirenz plasma concentrations which predicted neuropsychiatric toxicity, we did not observe a direct significant relationship between genotypes and neuropsychiatric toxicity but rather a tendency. All patients homozygous for CYP2B6*6 allele, except one experienced neuropsychiatric symptoms. Previous studies demonstrated association of CYP2B6*6 with early discontinuation of efavirenz-containing regimens mainly due to neurotoxicity [41,60,61] whereas others failed to demonstrate direct associations between *CYP2B6* genotypes. Similar to our finding a recent study in South African population reported a strong association of efavirenz plasma concentration and a tendency of CYP2B6*6 genotype with risk of developing neuropsychiatric symptoms [42]. Our findings reveal indirect association of *CYP2B6* genotype with efavirenz related neuropsychiatric disorders, indicating the need for more association studies with larger sample size and power. On the other hand, even with reported involvement of p-glycoprotein in the drugs oral absorption and cerebral spinal fluid concentrations, polymorphism of *ABCB1* at neither c.4036 A/G (rs3842) nor c.3435C/T was associated with efavirenz neuropsychiatric toxicity, although a tendency to influence day 3 efavirenz concentrations and significant effect on week-8 by c.4036 A/G (rs3843) was observed. Similarly no effect of CYP2B6*11 genotype on neuropsychiatric symptoms was observed despites its association with efavirenz plasma concentration at day-3, week-2 and week-4. The observed allele frequency of CYP2B6*11 and *ABCB1 c*.*4036G* alleles is lower than CYP2B6*6 allele in Ugandan population and hence our study sample size may lack adequate power to determine the actual effect size and hence future studies with larger sample size are required.

Current HIV treatment guidelines recommend immediate initiation of ART in HIV patients diagnosed with TB co-infection. Based on the endemicity of TB-HIV co-infection, there is an anticipated increase in patients concomitantly treated with efavirenz and rifampicin particularly in sub-Saharan Africa. The findings of this study, which on one hand allay concerns of possible pooled neuropsychiatric effects of efavirenz and anti-TB drug related adverse drug reactions [36,37,59], are in synchrony with periods of adaption of both CDC and WHO guidelines by Uganda and other countries within the African region. Our study may have important clinical implications indicating no effect of concomitant rifampicin therapy in HIV patients with respect to efavirenz associated CNS toxicity. Ideally, neuropsychiatric toxicity should be assessed against CSF efavirenz concentrations, which was not possible to do in the present study. However, results of this study indicate the relationship between *CYP2B6* and *ABCB1* genetic polymorphisms with efavirenz plasma concentrations.

## Conclusion

Efavirenz related neuropsychiatric symptoms are frequent and common among Ugandan HIV patients receiving ART. Having higher plasma efavirenz concentration, which is predicted by *CYP2B6* genotype, significantly increases the likelihood of developing neuropsychiatric symptoms in HIV patients. Therapeutic drug monitoring or *CYP2B6* genotyping practice in HIV clinics during early initiation of efavirenz therapy is encouraged to identify patients susceptible for neuropsychiatric disorders for optimal dosage adjustment. The finding of similar efavirenz concentrations and neuropsychiatric toxicity profiles in recipients of efavirenz with rifampicin or without rifampicin encourages concomitant clinical use of the two drugs among HIV/TB patients in Uganda and similar populations.

## Competing interests

The authors declare that they have no competing interests.

## Authors’ contributions

JM, EA: conceived and led the study, conducted data analysis and write the paper. JM: performed the genotyping and efavirenz plasma concentration determination. AO, NN, HL, DS: supervised neuropsychological assessments and scoring. JO, PW, LG: participated in the design of the study and overall management. All authors read and approved the final manuscript.

## Pre-publication history

The pre-publication history for this paper can be accessed here:

http://www.biomedcentral.com/1471-2334/13/261/prepub
